# Picoliter Droplet Generation and Dense Bead-in-Droplet Encapsulation via Microfluidic Devices Fabricated via 3D Printed Molds

**DOI:** 10.3390/mi13111946

**Published:** 2022-11-10

**Authors:** Tochukwu D. Anyaduba, Jonas A. Otoo, Travis S. Schlappi

**Affiliations:** 1 Keck Graduate Institute, Riggs School of Applied Life Sciences, Claremont, CA 91711, USA; 2 Abbott Rapid Diagnostics, 4545 Towne Center Ct, La Jolla, San Diego, CA 92121, USA

**Keywords:** microfluidics, picoliter droplets, rapid prototyping, bead encapsulation, 3D printing

## Abstract

Picoliter-scale droplets have many applications in chemistry and biology, such as biomolecule synthesis, drug discovery, nucleic acid quantification, and single cell analysis. However, due to the complicated processes used to fabricate microfluidic channels, most picoliter (pL) droplet generation methods are limited to research in laboratories with cleanroom facilities and complex instrumentation. The purpose of this work is to investigate a method that uses 3D printing to fabricate microfluidic devices that can generate droplets with sizes <100 pL and encapsulate single dense beads mechanistically. Our device generated monodisperse droplets as small as ~48 pL and we demonstrated the usefulness of this droplet generation technique in biomolecule analysis by detecting Lactobacillus acidophillus 16s rRNA via digital loop-mediated isothermal amplification (dLAMP). We also designed a mixer that can be integrated into a syringe to overcome dense bead sedimentation and found that the bead-in-droplet (BiD) emulsions created from our device had <2% of the droplets populated with more than 1 bead. This study will enable researchers to create devices that generate pL-scale droplets and encapsulate dense beads with inexpensive and simple instrumentation (3D printer and syringe pump). The rapid prototyping and integration ability of this module with other components or processes can accelerate the development of point-of-care microfluidic devices that use droplet-bead emulsions to analyze biological or chemical samples with high throughput and precision.

## 1. Introduction

Droplet microfluidics uses devices with channels dimensions tens or hundreds of microns wide to generate and manipulate discrete µL or less volumes. Dividing a sample of interest into fL to µL scale volumes reduces reagent usage, increases the sensitivity of chemical analyses, and provides enhanced control over reagent delivery, mixing, and chemical interactions [1]. There are many applications of droplet microfluidics in chemistry, biology, and biomedical engineering, such as therapeutic agent delivery, biomedical imaging, biomolecule synthesis, diagnostic chips, drug discovery, cell culture, biochemical characterization, and single cell analysis [2]. The implementation of droplet microfluidics in these applications are accomplished through lab-on-a-chip devices. These lab-on-a-chip devices may require droplet manipulation processes such as mixing, fission and/or fusion, sorting, and transportation of droplets [3,4], which can be accomplished via electrowetting, magnetic actuation, dielectrophoresis, surface acoustic waves, optical methods, or thermal methods [3,4,5,6,7]. However, due to the complicated processes used to fabricate channels that are tens or hundreds of microns wide, most droplet microfluidic methods are limited to research in laboratories with cleanroom facilities and complex instrumentation (e.g., photolithography with silicon wafers [8,9,10] or wet etching [11,12,13]). The few droplet generation technologies commercially available for diagnostic use are expensive ($89 k–$100 k for an instrument and $24–$240 per disposable cartridge) and not integrated with other assay steps such as chemical reaction incubation and droplet analysis [14].

To make the droplet generation process simpler, less time-intensive, and less expensive, many innovative methods have been created. Some researchers have used glass capillaries to generate pL-scale droplets. For example, Li et al. bonded microscope glass slides to pulled glass capillaries to generate monodisperse multiple emulsions [15], Gu et al. created and manipulated pL droplets for single cell assays with a 75 µm fused-silica capillary [16], and Li et al. used an asymmetrical beveled capillary to generate pL to nL droplets and execute a digital PCR assay [17]. While the instrumentation costs for these devices are lower than for photolithography, devices made from glass capillaries are difficult to integrate into other upstream or downstream modules and not amenable to rapid prototyping due to the intricate procedures for fabricating capillaries <1 mm in diameter. Other groups rely on micromachining to generate droplets, such as direct milling of polycarbonate [18,19] or micromachining in PMMA [20]. These methods have demonstrated consistent and controllable droplet generation; however, the droplet sizes are large (>1 nL) or when ply-sized droplets are achieved, a centrifuge is needed to create the droplets in a reaction tube, which precludes its ability to be integrated into other microfluidic modules [20].

3D printing is now commonly used to create molds for PDMS devices, which eliminates the need for cleanroom facilities, photolithography, or etching and enables rapid prototyping and fabrication [21,22,23,24]. Researchers have also used 3D printers to build monolithic devices out of resin for droplet generation, albeit with larger channel dimensions and therefore larger droplets (>1 nL) [25,26,27,28,29,30,31]. Picoliter-scale droplets are important for several applications, such as increasing the precision, sensitivity and dynamic range of digital PCR [32], or preventing cross contamination and target dilution in single cell analysis [16,33]. The small channel sizes required for pL-scale droplets are typically fabricated with complex processes insides a cleanroom, usually photolithography [8,9,10], and have not been made with 3D printed molds or 3D printed monolithic devices. The methods described above have significantly advanced droplet generation for the picoliter scale via photolithography or glass capillaries, and the nanoliter scale via 3D printing; however, there remains a need for <100 pL droplet generation from a rapid prototyping method (e.g., 3D printed molds) that can be easily integrated into other sample preparation, analysis, and detection modules.

An important area of investigation in droplet microfluidics are methods that encapsulate a single bead in a droplet (BiD). These BiD platforms have enabled exciting advancements in biomedical research and diagnostics, including genome sequencing [34], enzyme evolution and screening [35,36], detecting rare genetic mutations [37,38] single cell analysis [39], and molecular diagnostics [40]. While these devices have high throughput and multiplexing capabilities, they are limited to laboratories with sophisticated instrumentation for photolithography and bead encapsulation. Additionally, they have shown Poisson or better distributions of BiDs for particles with a similar density to water, such as gel particles [41], polystyrene beads [42,43,44], agarose beads [39], or biological cells [39,44,45]. Particles with a higher density than water sediment to the bottom before being encapsulated in droplets and cause the first fraction of droplets to have more than 1 bead per droplet and the remaining fraction to not have any beads. To use beads of varying densities in BiD platforms, this sedimentation effect must be overcome.

The purpose of this work is to overcome current limitations of droplet microfluidic devices by creating a droplet generation device with the following features: (i) a simple and inexpensive fabrication process that is amenable to rapid prototyping and integration with other modules, (ii) droplet volumes <100 pL, and (iii) the ability to encapsulate dense beads in aqueous droplets with a Poisson-like distribution. We found that using 3D printing to create a mold instead of photolithography or etching is a suitable fabrication method to accomplish this purpose. Our device generated monodisperse droplets as small as ~48 pL and we demonstrated the usefulness of this droplet generation technique in biomolecule detection by quantifying nucleic acids via digital loop-mediated isothermal amplification (dLAMP). We also designed a mixer that can be integrated into a syringe to overcome dense bead sedimentation and found that the BiD emulsions created from our device had less than 2% of the droplets populated with more than 1 bead when the average input concentration was 0.15 beads/droplet, in line with Poisson statistical projections. This study will enable researchers to create devices that generate pL-scale droplets and encapsulate dense beads with inexpensive and simple instrumentation (3D printer and syringe pump). The rapid prototyping and integration ability of this method can accelerate the development of point-of-care microfluidic devices that generate droplet-bead emulsions and analyze samples with high throughput and precision.

## 2. Materials and Methods

### 2.1. Device Fabrication

**Figure 1:**
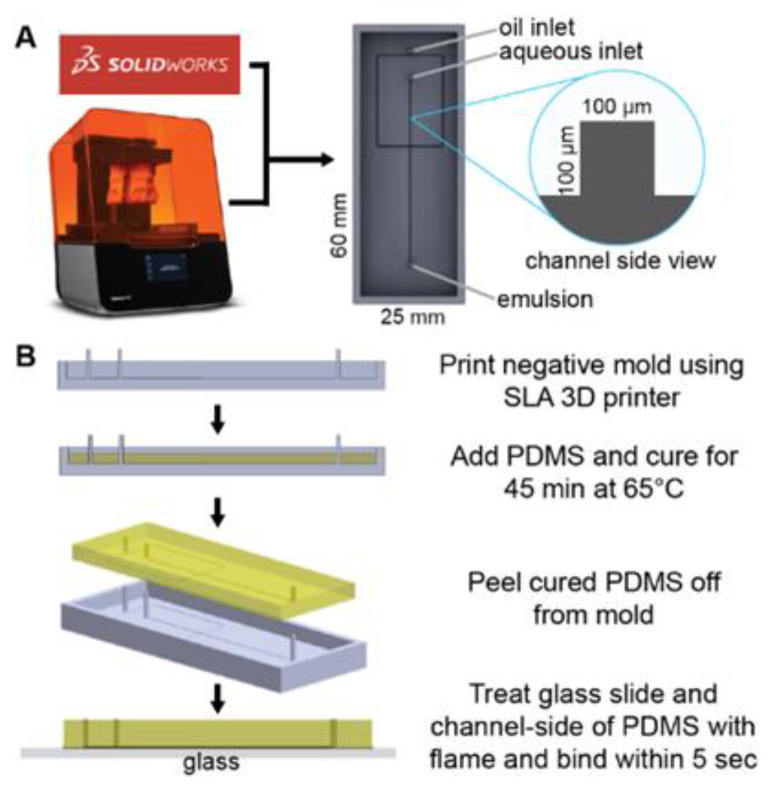
Microfluidic device design and fabrication. (**A**) A solid master mold was designed with Solidworks CAD software and printed with FormLabs Form3 SLA printer. (**B**) PDMS device fabrication process.

3D models of the master molds were designed using SolidWorks CAD software (Dassault Systems, Velizi-villacoublay, France) to have flow channel dimensions of 100 µm × 100 µm and inlet/outlet ports of 750 µm (Figure 1A). Stereolithography (SLA) files were prepared for 3D printing by orienting them at a 45° angle and avoiding cups and overhangs in Form Labs’ Preform software. The models were then printed using the Form3 SLA 3D printer (Form Labs) in Clear resin (FLGPCL04) at a layer thickness of 25 µm. The printed master molds were thoroughly cleaned with isopropyl alcohol to remove excess resin, then UV-cured for 30 min.

To make polydimethylsiloxane (PDMS), SYLGARDTM 184 Silicone Elastomer Base and SYLGARDTM 184 Silicone Elastomer Curing Agent (Dow Corning, Midland, MI, USA) are combined at 10:1 *w/w* ratio to make up ~3 gm needed to fill each mold. Prior to pouring the mixture into the mold, it is degassed in a Cole Parmer Diblock oven at room temperature until no bubbles can be seen in the PDMS mixture. After filling the molds with the degassed PDMS, the degassing process is repeated to ensure complete filling of the corners of the channels before curing at 65 °C for 45 min. Once cured, the PDMS is gently peeled from the master mold and bonded onto glass microscope slides (Amscope BS-72P 100S-22) after surface activation using flame treatment as an alternative to oxygen plasma bonding [46] (Figure 1B). The device is then placed in an 85 °C oven overnight to allow the PDMS to harden. Next, the devices are examined for binding strength of the PDMS by gently prying at them. They are also checked for channel dimensions under a microscope. A ± 10% tolerance is allowed for the channel widths measured from micrographs prior to the attachment of the flow tubing (Scientific Commodities, Lake Havasu City, AZ, USA, BB31695 PE/3). The tubing is attached to the chip by plumbing them into the inlet and outlet ports, making sure to leave a clearance space between the tubing nozzle and the slide surface. The tubing is further held in place using cold weld steel-reinforced epoxy (JB Weld, Marietta, GA, USA).

### 2.2. Droplet Generation

Droplets were generated using the designed flow-focusing PDMS microfluidic devices described above. The oil phase consisted of mineral oil (Sigma Aldrich M3516-1L), 0.1 wt% Triton X-100 (Fisher Scientific, Waltham, MA, USA), and 3 wt% ABIL EM 90 (Evonik, Essen, Germany), and was pumped at various volumetric flow rates (20, 25, 50, 75, 100 µL/min). The aqueous phase (DI water) was maintained at a volumetric flow rate of 1 µL/min. The oil and aqueous phases were pumped to an intersection in the device by syringe pumps (KD Scientific, Holliston, MA, USA), at which point droplets were generated and subsequently collected from the outlet in Eppendorf tubes. A fraction of the droplets were imaged using confocal imaging (Leica SP5, Wetzlar, Germany) and the respective planar areas of the droplets were deduced using ImageJ software after segmentation processing. The spherical diameter of each droplet is calculated from the deduced area.

### 2.3. Droplet Digital Loop-Mediated Isothermal Amplification for DNA Quantification

*Lactobacillius acidophilus* (L. acid.) obtained from MicroKwik vials (Carolina Biological Supply, Burlington, NC, USA) was cultured in de Man, Rogosa and Sharpe (MRS) agar formulated in-house using Millipore-Sigma formulation (CCW4691). The QuickExtractTM one-step DNA extraction kit (Lucigen, Middleton, WI, USA) was used to extract DNA from the colonies. Extracted genomic DNA was quantified via absorbance measurements from a Nanodrop One instrument (ThermoFisher Scientific, Waltham, MA, USA) and diluted in nuclease-free water to concentrations ranging from 0 to 9.5 × 106 copies/mL.

LAMP master mix was prepared with final concentrations of 1× isothermal amplification buffer (New England Biolabs, NEB), 8 mM of MgSO4 (NEB), 1.4 mM dNTPs (NEB), 320 U/ mL Bst 2.0 WarmStart polymerase (NEB), primer mix, and 1× SybrGreen (Life Technologies). The primer mix was designed in-house to target the *L. acidophilus* 16S rRNA gene and consisted of 1.6 µM each of forward inner primer (CTGCACTCAAGAAAAACAGTTTCCGAGTCTGATGTGAAAGCCCTC) and backward inner primer (AAGAGGAGAGTGGAACTCCATGTGAGACCAGAGAGCCGCCTT), 0.2 µM each of forward outer primer (TAAAGCGAGCGCAGGC) and backward outer primer (CCTCAGCGTCAGTTGC), 0.4 µM each of forward loop primer (GCAGTTCCTCGGTTAAGCC) and backward loop primer (ATGCGTAGATATATGGAAGAACACC) (Integrated DNA Technologies, Clarville, IA, USA). *L. acid* DNA dilutions were added to LAMP master mix to yield final concentrations of 0, 1.0·× 107, 2.5·× 107, 5.0 × 107, 4.0·× 108 DNA copies/mL (quantified by Nanoquant absorbance measurements). Four replicates of each dilution (10 uL/well) were amplified at 68 °C for 60 min using a LightCycler® 96 Instrument (Roche, Basel, Switzerland) as positive controls.

The LAMP mix + *L. acid* DNA samples were infused into a droplet generation device as described in “Droplet Generation”, with oil flow rate 75 µL/min and aqueous flow rate 1 µL/min. Droplets from the microfluidic devices were collected in amber SepCap vials (Thermoscientific, Waltham, MA, USA C4015-99) and incubated at 68°C for 60 min using a Multi-Therm shaker (Benchmark Scientific, Sayreville, NJ, USA). After incubation, the droplets were imaged using a Leica SP5 confocal microscope, and images were analyzed with Image J to determine the relative fluorescence intensity (RFI) of each droplet. A threshold was determined by computing $\mu_{NTC}+3\cdot \sigma_{NTC}$, where $\mu_{NTC}$ is the mean and $\sigma_{NTC}$ is the standard deviation of the RFI of the 0 cop/mL sample droplets. Droplets with RFI greater than the threshold were classified as positive while the droplets less than or equal to the threshold are classified as negative. One can then use Poisson statistics with the number of positive and negative droplets to calculate a concentration for each sample [47].

### 2.4. Bead Mixer

A blind hole with a diameter of about 9 mm was drilled into the side of a 3 mL plastic syringe (CareTouch, Westminster, CO, USA) at the 0.5 mL mark. A small DC motor with a plastic impeller which was originally designed for a bead-beating sample preparation device (Claremont Bio 01.340.48 OmniLyse® Kit) was retrieved and carefully positioned into the syringe through the blind hole. The motor with the impeller was affixed to the syringe with cold weld steel-reinforced epoxy (JB Weld, Marietta, GA, USA) such that the blind hole was completely sealed and airtight. The epoxy was allowed to set for 48 to 72 h. The impeller mixer was powered by a 1.5 V DC power supply (SI, Figure S1).

### 2.5. Bead-in-Droplet Emulsions

Hard shell Polymethyl Methacrylate (PMMA) beads (PolyAn Microshperes Po-105 00 020 and Alpha Nanotech colloidal PMMA) of 20 µm in diameter were used in the bead encapsulation experiment. A mixture of the beads and 0.1 %*v*/*v* Tween 20 in nuclease-free water at working concentrations of 0.15, 0.2 and 0.3 beads/droplet (λ) were used as the dispersed phase for the experiments. A mixture of mineral oil (Sigma Aldrich-M3516-1L), 0.1 wt% Triton X-100 (Fisher Scientific, Waltham, MA, USA) and 3 wt% ABIL EM 90 (Evonik, Essen, Germany) was used as the continuous phase. The dispersed phase (bead suspension) was aspirated into a modified syringe and loaded onto a syringe pump (KD Scientific, Holliston, MA, USA, KDS100). A 1.5 V DC power supply was connected to the mixer to keep the beads solution homogenous. The continuous phase was put into a 10 mL plastic syringe (CareTouch, Westminster, CO, USA) and loaded onto a syringe pump. The continuous and dispersed phases were introduced into the droplet generation device using syringe pumps at flow rates of 30 µL/min and 1–7 µL/min, respectively. A period of about 5 min was allowed for the cartridge to be primed and for the droplet generation to be stabilized. The droplets were collected from the cartridge into 1 mL amber SepCap vials (Thermoscientific, Waltham, MA, USA, C4015-99). The excess oil from the continuous phase was poured off and the droplets were put onto a microscope slide and mounted onto a microscope (Omax microscope 3152102) for imaging. Micrographs of the droplets were taken using the Amscope microscope camera md35 and Amsocpe software version 4.

### 2.6. Image Analysis

The images were opened in Image J. The scale was set according to the scale bar on the images and the unit was set to µm. The images were converted to 8-bit gray scale images and speckles and noise were filtered from the images. The threshold of the images was adjusted to convert them to binary images. The images were converted to mask to invert the black to white, making the droplets appear white. The droplets were then analyzed to calculate the area of each droplet. The diameter and volume of each droplet were calculated from the area of the droplets. The droplets containing beads were manually counted and the number of beads in each droplet was recorded. The data were compiled in Excel (Microsoft Office) and parsed into Python 3.0 for further analysis and visual presentation.

## 3. Results and Discussion

### 3.1. Picoliter-Scale Droplet Generation

The physics of droplet generation via flow focusing has been well documented with theory and experiments showing an inverse logarithmic relationship between Capillary number ($Ca=\mu_{ave}\left(2Q_{o}+Q_{w}\right)/\sigma hw$) and non-dimensionalized droplet diameter, $D_{d}/D_{h}$, where $\mu_{ave}$ is the average viscosity of the two fluids, $Q_{o}$ is the oil flow rate, $Q_{w}$ is the water flow rate, σ is the surface tension, h is the channel height, w is the channel width, $D_{d}$ is the diameter of the droplet, and $D_{h}$ is the hydraulic diameter of the channel, 2hw/(h+w) [48,49]. These flow focusing studies demonstrate that <100 pL droplets can theoretically be generated with Ca>0.001 (faster flow rates ($Q_{o}$,$Q_{w}$) relative to channel dimensions (h,w)) and 144 µm > $D_{h}$ > 39 µm, or with Ca<0.001 (slower flow rates ($Q_{o}$,$Q_{w}$) relative to channel dimensions (h,w)) and 14 µm $<D_{h}$ < 39 µm [48] (SI, Section S2). Experimentally, the authors test devices with maximum channel heights of 27 µm [48] or widths of 71 µm [49]. In these studies and others [8,9,10,11,12,13], pL droplets are generated by using small channel widths (<100 µm) facilitated by photolithographic processes in cleanrooms. As our objective was to develop a device that generates pL droplets without complex fabrication processes, we were limited to the channel widths 100 µm or greater that an SLA 3D printer is capable of printing in a mold. Therefore, our device design would need to be in the Ca > 0.001 regime with faster flow rates relative to channel dimensions.

**Figure 2:**
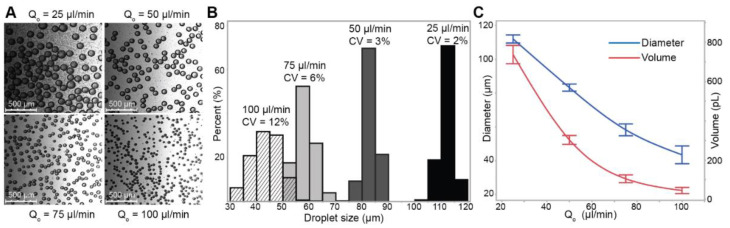
Picoliter-scale droplet generation. (**A**) Micrograph of the droplets retrieved from microfluidic cartridge outlet. (**B**) Droplet diameter distribution and CV at each flow condition. (**C**) The droplet diameter changes with volumetric flow rate of the oil phase. The volumetric flow rate of the aqueous phase was kept constant at 1 µL/min.

With the limits on our device’s physical features established, we 3D printed a mold and made a PDMS cast of 100 µm channel width and 100 µm channel height without a cleanroom, photolithography processes, or complex instrumentation (Figure 1). We chose oil and water flow rates such that the droplet generation device would have Ca≫ 0.001, with $Q_{o}$ = 25 to 100 µL/min and $Q_{w}$ = 1 µL/min (SI, Section S2), which resulted in droplets of diameters 45 to 112 µm (48 to 736 pL) (Figure 2). The droplets generated from this device are monodisperse (Figure 2B, coefficient of variation (CV) from 2–12%), which is in the range of droplets generated from other devices [50,51]. As expected, there is an inverse power relationship between droplet volume and oil flow rate [49], showing that devices fabricated with 3D printed molds give similar consistency and expected performance at the picoliter scale as devices made with photolithography in a cleanroom. Because this device is made from a 3D printed mold, researchers can iterate prototypes rapidly without undergoing the time and resource-consuming processes of photolithography; additionally, the droplet generation module can be part of a larger 3D printed mold that includes modules for executing other upstream or downstream assay processes.

### 3.2. Droplet Digital Loop-Mediated Isothermal Amplification

To explore the utility of this droplet generation device in molecular diagnostic applications, droplet digital loop-mediated isothermal amplification (ddLAMP) was performed to detect and quantify a DNA target. Digital LAMP is an emerging nucleic acid (NA) amplification method that can quantify the NA concentration of a sample with high accuracy and precision, even in the midst of temperature, reaction time, or imaging variance [52]. NA quantification via dLAMP is useful in several applications, such as viral load measurements for HIV [53], hepatitis C virus genotyping [54], and rapid antibiotic susceptibility testing [55]. Current dLAMP methods partition the sample into pL to nL droplets with microfluidic devices made using photolithography [56,57], wet etching [52,53,54,55], or fused-silica capillaries [58]. Our droplet generation device made from a 3D printed mold could make dLAMP more accessible by eliminating the need for complex facilities or instruments and enabling integration with other amplification or detection modules.

**Figure 3:**
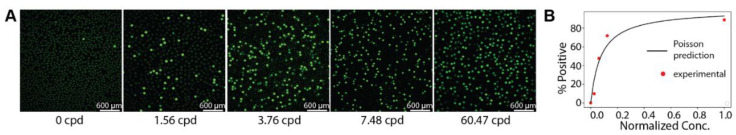
Droplet digital LAMP. (**A**) Post-amplification micrographs of droplets. (**B**) Agreement between Poisson predicted positive droplet percentage and experimental data.

We tested the feasibility of encapsulating LAMP reagents with target DNA and primers into droplets with our device (Materials and Methods). After generation, the droplets were incubated at 68 °C for 60 min for amplification of DNA via LAMP and SybrGreen fluorescence was measured to indicate the presence or absence of amplification product within each droplet (Figure 3A). Five DNA dilutions were tested, and the positive droplet percentage was plotted against the prediction from Poisson statistics (Figure 3B), assuming a 10% LAMP efficiency and 300 pL droplet volume (SI, Section S3).

### 3.3. Dense Bead-in-Droplet Emulsions

Interest in using microparticles as delivery systems in various technologies has been widely researched, especially in combination with microdroplets for biological applications [59,60,61,62]. This is due to the high surface-to-volume ratio and the ease of immobilizing biorecognition molecules on them, as well as the potential for compartmentalized single-molecule assays [63,64,65]. Single particle encapsulation in droplets, however, faces two major challenges: sedimentation due to particle density [62], and mechanistic single particle encapsulation [41,66].

Particle density poses a challenge when loading microparticles into encapsulation devices because the higher density particles (>1 gm/mL) sediment in the syringe and delivery tubing, causing nonhomogeneous distribution of microparticles in droplets (SI, Figures S1A and S2). This can be solved by the dissipation of the bead density by suspending them in denser fluids such as glycerol [62]; however, such fluids may not be compatible with the intended bio-application. For example, glycerol at 50% *v*/*v* inhibits NA amplification, thereby defeating the purpose of using microbeads for NA applications (SI, Figure S4). To circumvent this challenge, researchers used gel beads with similar density to water, which ensured a binary distribution of beads in the droplets [65,67,68,69]. However, this method is time-consuming, requiring a particle velocity of ~50 µm/h [41] to achieve single-particle encapsulation; furthermore, some multiplexed nucleic acid detection methods are not compatible with beads made in gel form [70,71,72].

Price et al. presented a potentially simple solution by exploiting the sedimentation potential of the beads using a hopper system [62]. They, however, showed that it took 0.8 h (17 µm Tetangel resin beads) and 3.8 h (2.8 µm magnetic beads) for bead introduction before achieving single bead encapsulation. Kim et al. successfully developed a pneumatic system which is capable of trapping and releasing beads, thus creating a deterministic encapsulation of a defined number of beads per droplet [62]. This system is not simple to develop or operate, thus, unfit for low-cost point-of-care devices that can integrate with other modules.

**Figure 4:**
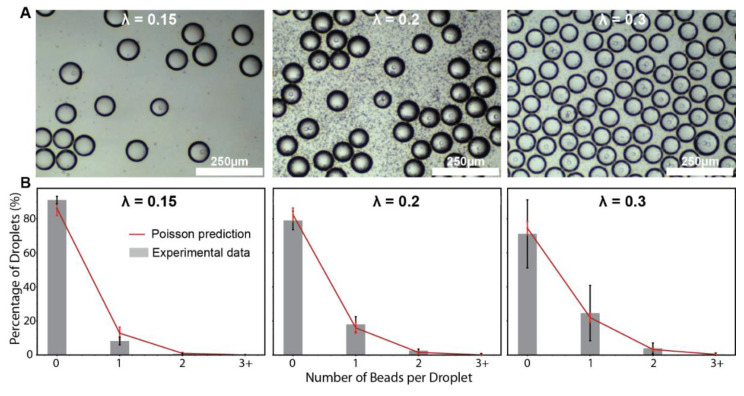
Dense bead in droplet (BiD) encapsulation at varying bead concentrations (λ = 0.15, 0.2, 0.3). (**A**) Microscope images of BiD. (**B**) Poisson-predicted bead in droplets distribution in comparison to the observed experimental data.

Our goal was to present a simple, easy-to-fabricate method to encapsulate single dense beads in droplets that can be used for further downstream analysis. It is important to encapsulate single beads as opposed to multiple beads to avoid cross-contamination or confusion of which target molecule or bead is in the droplet. The idea is to vertically orient the syringe pump while keeping the beads suspended by mechanical agitation (which prevents loss of beads due to sedimentation in the flow tubing and in the syringe) (Figure S1B), then pump the contents directly into the droplet generation cartridge (Figure 1B). Using this principle, we set up bead encapsulation with the droplet generation device such that λ ≈ 0.15, 0.2 and 0.3 beads/droplet, where λ represents the average number of beads per droplet input into the device (Figure 4). We observed that our dense bead encapsulation method agreed well with Poisson predictions (Figure 4B). Importantly, the droplet generation device resulted in <2% of droplets containing more than 1 bead at λ ≈ 0.15, <4% of droplets containing more than 1 bead at λ ≈ 0.2, and <6% of droplets containing more than 1 bead at λ ≈ 0.3.

## 4. Conclusions

Using design principles from droplet microfluidic device literature, we designed and developed a microfluidic device fabricated without complex equipment or cleanroom facilities that can generate sub-100 pL droplets and encapsulate dense beads with a Poisson-like distribution. Because the device is made from a 3D printed mold, researchers can iterate prototypes rapidly without undergoing the time and resource-consuming processes of photolithography; additionally, the droplet generation module can be part of a larger 3D printed mold that includes modules for executing other upstream or downstream assay processes, such as sample preparation, NA amplification, or single cell analysis.

While simple instrumentation was used to fabricate the microfluidic device, we still needed a syringe pump for operation of the device to generate consistent and controlled droplet sizes. Further improvements need to be made to our design to make it more amenable to point-of-care settings, such as a pumping lid [73], or other equipment-free pumping mechanisms [74]. Another limitation is that due to the 3D printer’s minimum channel dimension (~100 µm), the lowest droplet diameter achieved was 45 µm (48 pL). Lower sizes could be possible in the future with the next generation of 3D printers that print channels down to 15 µm [75].

Other microfluidic devices have encapsulated beads in a non-random distribution and thus have a much higher percentage of droplets with a single bead [41,43], though the beads in those studies have a similar density to water. While the phenomenon for the non-random distribution is unexplained, similar designs could potentially be used with the dense bead mixing method studied here for higher percentages of droplets with single beads. In its current form, this device enables research and innovation into assays or methods that need to use beads with a density greater than water and thus overcome the sedimentation effect, such as PMMA or magnetic beads. Because it can easily be printed and combined with others as part of a larger device, microfluidic sorting mechanisms can also be used to concentrate the beads downstream if desired [76].

Future research directions from this work can include: eliminating the need for a syringe pump for <100 pL droplet generation, adapting the device to other biological assay applications beyond digital LAMP, beating Poisson encapsulation statistics for dense beads to reduce the waste of empty droplets, or adapting the BiD method for tagging multiple biomarkers. Due to the simple instrumentation used, this work enables rapid prototyping for a variety of biological applications of droplet microfluidic devices and dense bead encapsulation.

## Data Availability

The data presented in this study are available in this article and Supplementary Materials.

## Supplementary Materials

The following supporting information can be downloaded at: https://www.mdpi.com/article/10.3390/mi13111946/s1, Section S1: Syringe with mixer to overcome sedimentation effect of dense beads; Section S2: Capillary number calculations for picoliter-scale droplet generation design; Section S3: Poisson prediction of positive droplet percentage; Section S4: Effect of Glycerol on LAMP Amplification; Section S5: Pitalls of 3D Printing Fabrication of Microfluidic Cartridges. Figure S1: (A) Tube connecting syringe containing bead suspension to the droplet generation cartridge; red arrow shows region of bead sedimentation. (B) Syringe design for mechanical resuspension and homogenization of dense particles for vertical delivery. The DC motor is powered using a 3V battery. Figure S2: Without the syringe mixer in Figure S1, bead sedimentation happens in the syringe and tubing, leading to the encapsulation of multiple beads per droplet. Figure S3: Denser fluids, such as glycerol, may improve bead buoyancy but it inhibits LAMP amplification (blue trace vs. red trace). Bead Density = 1.18 g/ cm^3^, Glycerol Density = 1.26 g/cm^3^. Figure S4: Microcapillary lines imprinted by 3D printed mold. This is often due to printer-head misalignment that often occurred during prolonged prints. Figure S5: Micrograph showing curved vertices imprinted from 3D-printed mold. Figure S6: Irregularities in chamber dimensions due to myriad factors, including incompletely cured PDMS and build-up PDMS deposit due to mold reuse. Note that the displayed images contain channels designed to have widths of 50 and 100 µm. Figure S7: Frosted PDMS molded on improperly cleaned 3D printed mold. Figure S8: Image of final fabricated PDMS device and 3D printed mold.
