# Supplementary material for: Picoliter Droplet Generation and Dense Bead-in-Droplet Encapsulation via Microfluidic Devices Fabricated via 3D Printed Molds
Source: Micromachines (Basel). 2022 Nov 10;13(11):1946. doi: 10.3390/mi13111946 (PMC9695966; doi:10.3390/mi13111946)
Supplement: Supplementary file 1 [file micromachines-13-01946-s001.zip › micromachines-2015257-supplementary.pdf]

Picoliter droplet generation and dense bead-in-droplet encapsulation via microfluidic devices fabricated via 3D printed molds

Tochukwu D. Anyaduba[1,2], Jonas A. Otoo[1], Travis S. Schlappi[1,*]

[1] Riggs School of Applied Life Sciences, Keck Graduate Institute, 535 Watson Drive, Claremont, CA 91711, USA
[2] Abbott Rapid Diagnostics, 4545 Towne Center Ct, La Jolla, San Diego, CA 92121
* Correspondence: Travis_Schlappi@kgi.edu

Supplementary Information

<u>Section S1:</u> Syringe with mixer to overcome sedimentation effect of dense beads.

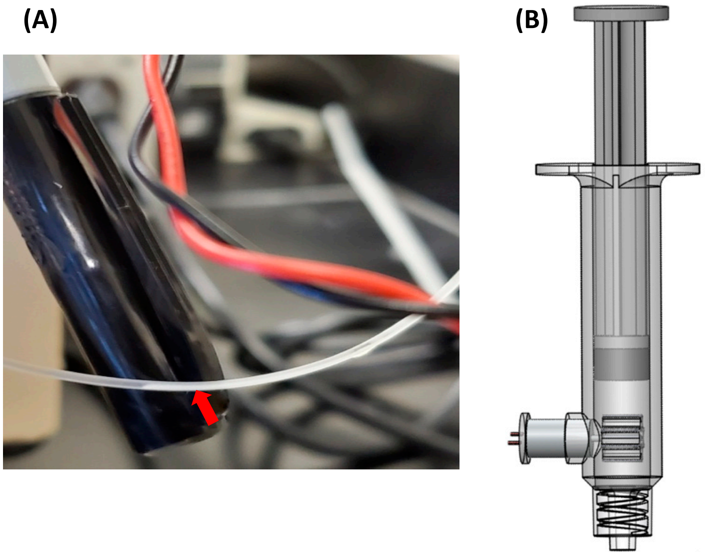

Figure S1. (A) Tube connecting syringe containing bead suspension to the droplet generation cartridge; red arrow shows region of bead sedimentation. (B) Syringe design for mechanical resuspension and homogenization of dense particles for vertical delivery. The DC motor is powered using a 3V battery.

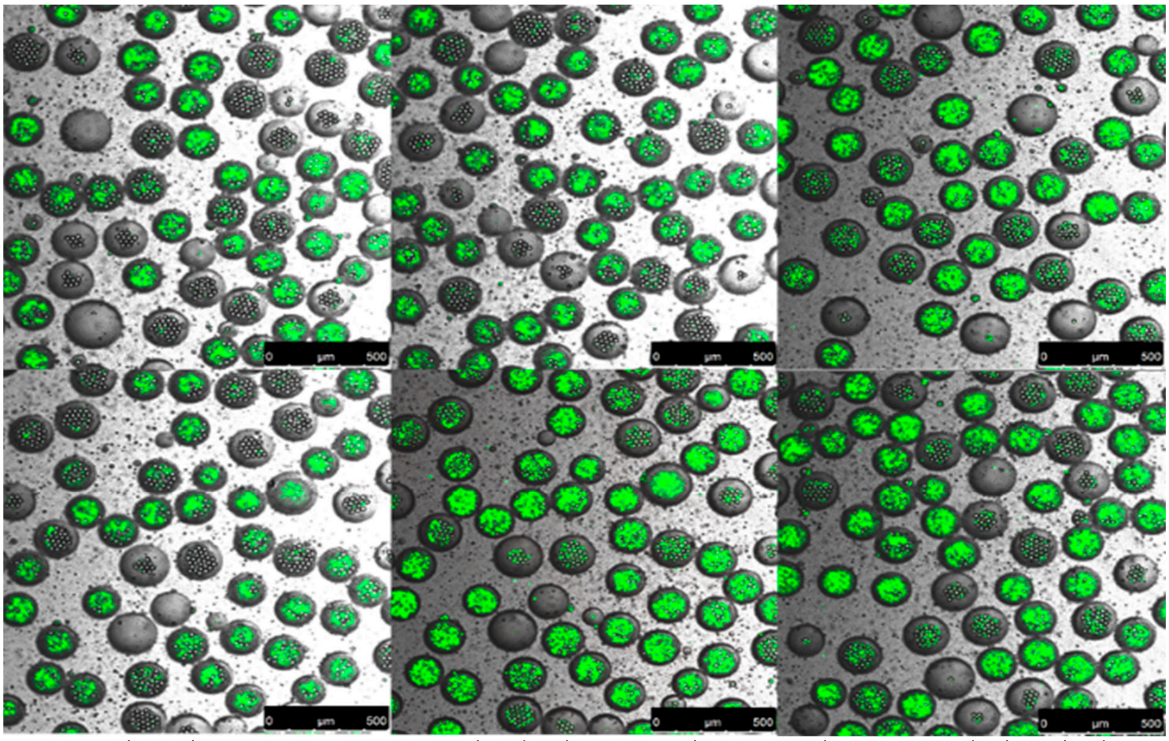

Figure S2. Without the syringe mixer in Fig. S1, bead sedimentation happens in the syringe and tubing, leading to the encapsulation of multiple beads per droplet.

Section S2: Capillary number calculations for picoliter-scale droplet generation design.

From Figure 5 of Reference [44], $\frac{r_d}{D_h} = 0.053 \cdot Ca^{-0.38}$, with r_d = droplet radius, D_h = hydraulic diameter, and Ca = capillary number. The range of $\frac{r_d}{D_h}$ studied in this work was 0.2 to 2. Using this equation, $2 > \frac{r_d}{D_h} > 0.73$ corresponds to $Ca < .001$, and $0.2 < \frac{r_d}{D_h} < 0.73$ corresponds to $Ca > .001$.

A 100 pL droplet has $r_d = \left(\frac{100pL}{\frac{4}{3}\pi}\right)^{1/3} = 28.8\mu m.$

Therefore, in the $Ca > .001$ regime, $0.2 < \frac{r_d}{D_h} < 0.73 \rightarrow D_h > \frac{28.8\mu m}{0.73} = 39.3\mu m$

$$\rightarrow D_h < \frac{28.8\mu m}{0.2} = 144\mu m.$$

In the $Ca < .001$ regime, $2 > \frac{r_d}{D_h} > 0.73 \rightarrow D_h < \frac{28.8\mu m}{0.73} = 39.3\mu m$

$$\rightarrow D_h > \frac{28.8\mu m}{2} = 14.4\mu m.$$

Capillary number calculation using Equation (6) from Reference [44]:

$$\mu_{ave} = \left(\frac{1}{2\mu_o} + \frac{1}{2\mu_i}\right)^{-1} = 1.766\ cp$$

$$Ca = \frac{\mu_{ave}(2Q_o + Q_i)}{\sigma h w_{or}} = 0.148$$

$$\mu_{oil} = 30cp; \mu_{water} = .91cp; Q_o = 100\ \mu L/min; Q_i = 1\ \mu L/min;$$

$$\sigma = 4mN/m; h = 100\mu m; w_{or} = 100\mu m$$

Section S3: Poisson prediction of positive droplet percentage

The input DNA concentrations as measured by NanoDrop were 0, $1.0{\cdot}10^7$, $2.5{\cdot}10^7$, $5.0{\cdot}10^7$, $4.0{\cdot}10^8$ DNA copies/mL. From previous digital LAMP experiments, we estimated that the LAMP efficiency compared to PCR for our LAMP primers was ~10% and the droplet sizes generated from the device were ~300 pL. With these estimations for LAMP efficiency and droplet size, we then used Equation (2) from Reference [43] to calculate the percentage of positive droplets expected at each input DNA concentration.

$$\frac{b}{n} = e^{-v\lambda} \rightarrow Pos\% = 1 - \frac{b}{n} = 1 - e^{-v\lambda}$$

where b = # of negative droplets, n = # of total droplets, v = droplet volume in mL, and λ = DNA concentration in copies/mL.

For example, at $2.5{\cdot}10^7$ copies/mL, Equation (2) predicts 53% of droplets will be positive:

$$Pos\% = 1 - e^{-v\cdot(\lambda\cdot eff)} = 1 - e^{-(3\cdot 10^{-7})\cdot(2.5\cdot 10^7)\cdot 0.1} = 52.8\%$$

Section S4: Effect of Glycerol on LAMP Amplification

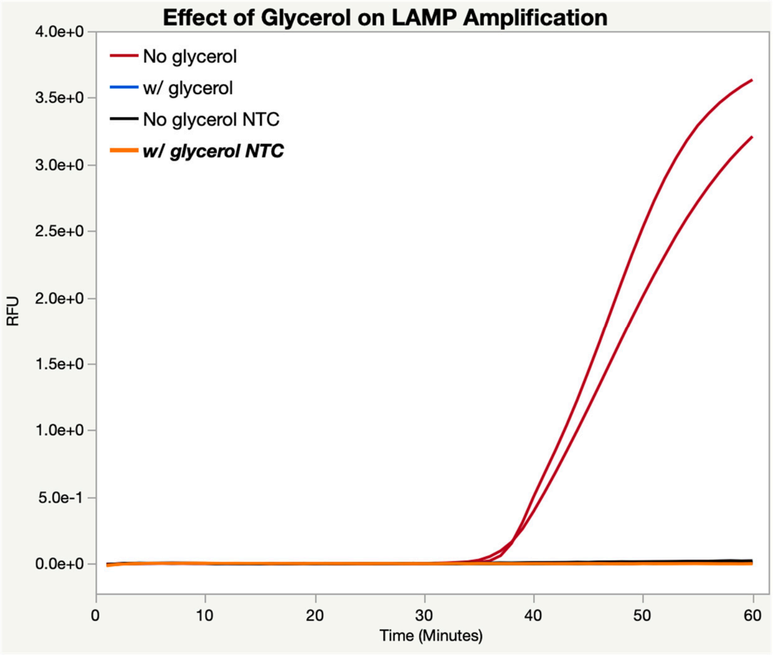

Figure S3. Denser fluids, such as glycerol, may improve bead buoyancy but it inhibits LAMP amplification (blue trace vs red trace). Bead Density = 1.18 g/ cm^3, Glycerol Density = 1.26 g/cm^3.

Section S5: Pitfalls Of 3D Printing Fabrication of Microfluidic Cartridges

The pitfalls of 3D printing fabrication of microfluidics cartridges fall under to categories.

1. Equipment-dependent Pitfalls

One of the challenges attendant to using 3D printing for microfluidics is the limitation of the printer's resolution. We found that despite the advancements in 3D printing, especially desktop SLA printers, it is difficult to print channels with widths less than 50 µm. More so, the printing of master molds at higher resolutions (for example, 25 µm layer height) forces the printer to run for prolonged hours, thus, giving room for misaligned prints. This misalignment creates micro-channels in the molded PDMS, thereby causing leakage in the final cartridge (Figure S4). Another consequence of the printer limitations is the difficulty in printing well-defined edges. This impacts flow focusing more as there is always a characteristic curvature at the flow junction (Figure S5).

2. Procedural Pitfalls

As shown in Figure S6, batch-to-batch variations in channel dimensions constitute a significant limitation of this system. This often comes from the post-print cleaning of master molds. Inadequately cleaned edges (especially around the flow channels) and other dead spaces may lead to increased channel dimensions once the left-over Stereolithographic (SLA) printer resin cures.

Variations in channel dimension also occur when PDMS get trapped in the edges of the printed molds. If the cured PDMS is not peeled off completely from the mold, there is always a risk of losing the channel wall definitions, which, in turn would affect the fluidic chip channel dimensions. More so, the idea behind the use of a 3D-printed mold is to encourage the reuse of the molds. However, with the build-up of residual PDMS on the mold, the channel dimensions continuously increase with continued reuse. This is solved by limiting the number of reusage of molds to 3 to 5 times.

Improperly cleaned molds also create a very rough finish on the molds which in turn are imprinted on the PDMS structures causing them to have frosted/ unclear appearance. In our experience, frosted PDMS casts do not bond properly onto the glass substrate (Figure S7). This is mitigated by carefully and properly cleaning the molds before reuse, to eliminate any residual debris from previous use.

If the PDMS is not completely cured, attempts to peel them off the mold creates irregularities in the channel dimension and definition. In most cases, the channel may become occluded, tapered, or collapse due to efforts to bind the molded PDMS on the glass substrates. This challenge is addressed by ensuring the PDMS is allowed enough time to cure properly. The pitfalls are mitigated in the final PDMS fabricated device when all the outlined precautions are taken (Figure S8).

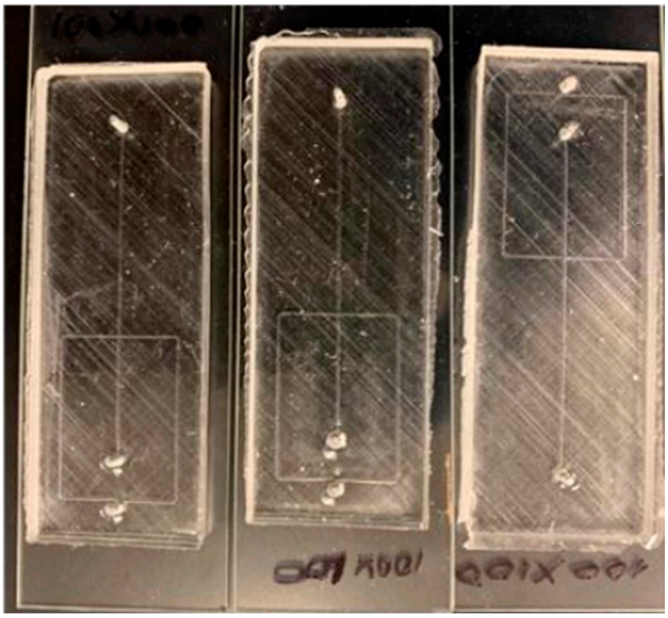

Figure S4. Microcapillary lines imprinted by 3D printed mold. This is often due to printer-head misalignment that often occurred during prolonged prints.

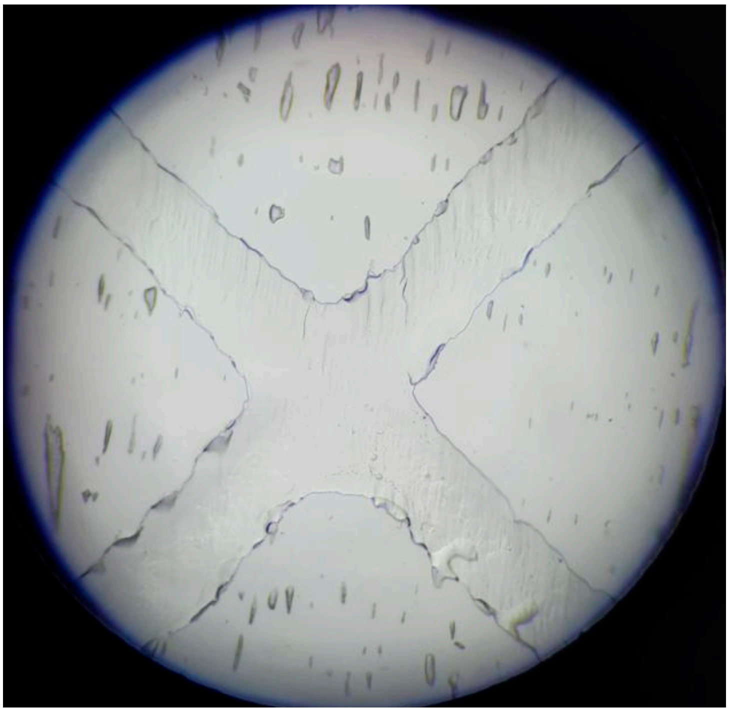

Figure S5. Micrograph showing curved vertices imprinted from 3D-printed mold.

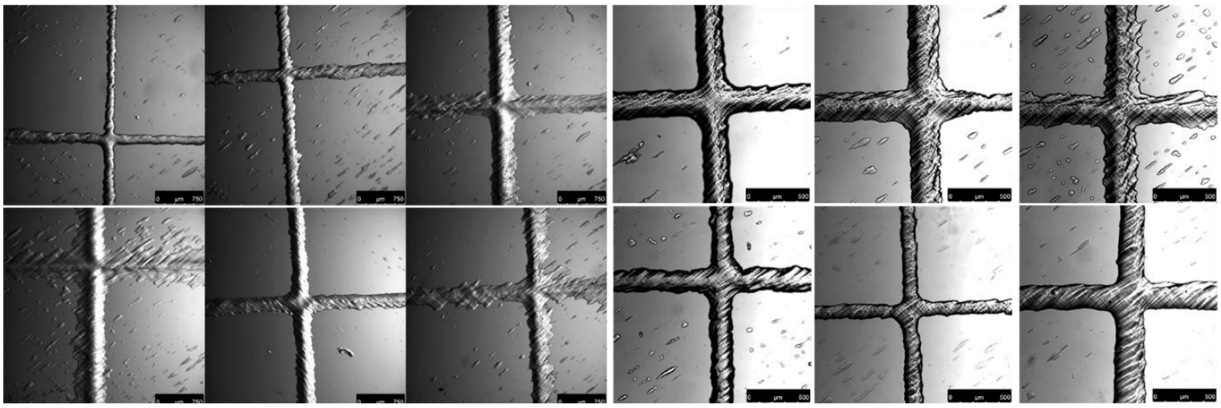

Figure S6. Irregularities in chamber dimensions due to myriad factors, including incompletely cured PDMS and build-up PDMS deposit due to mold reuse. Note that the displayed images contain channels designed to have widths of 50 and 100 µm.

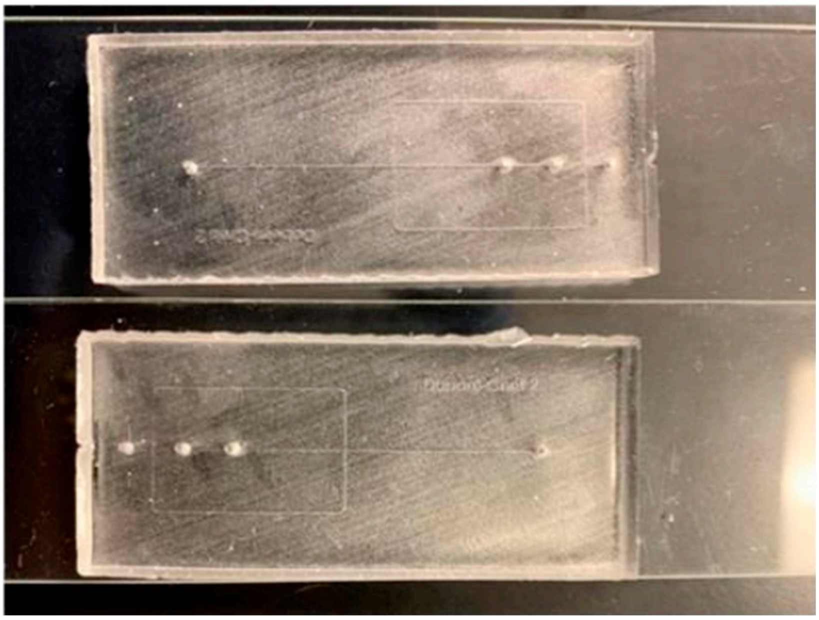

Figure S7. Frosted PDMS molded on improperly cleaned 3D printed mold.

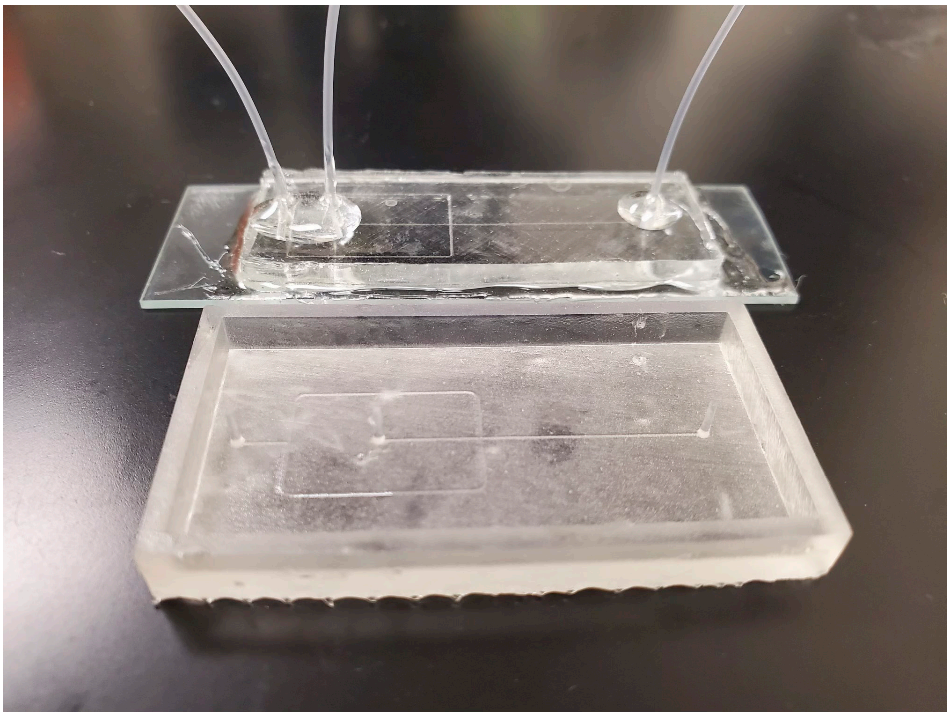

Figure S8. Image of final fabricated PDMS device and 3D printed mold
